# Effect of renal sympathetic denervation on ventricular and neural remodeling

**DOI:** 10.1007/s00059-018-4698-y

**Published:** 2018-04-12

**Authors:** L. Wang, G. Wei, L. Song, C. Li, F. Zhang, Y. Yang, C. Lu

**Affiliations:** 1 Tianjin First Center hospital, Clinical medical college of Tianjin Medical university, Tianjin, China; 2 Deparment of Caridiology, Tianjin First Center Hospital, 24 Fukang Road,Naikai District, 300192, Tianjin, China; 3 Department of Digestion, Tianjin First Center Hospital, Tianjin, China; 4 Department of Cardiology, Danzhou People’s Hospital, Danzhou, China

**Keywords:** Myocardial infarction, Heart attack, Sympathectomy, Cardiac remodeling, Experimental animal model, Myokardinfarkt, Herzinfarkt, Sympathektomie, Kardiales Remodeling, Experimentelles Tiermodell

## Abstract

**Background:**

This study assessed the therapeutic effects of renal sympathetic denervation (RDN) on post-myocardial infarction (MI) ventricular remodeling and sympathetic neural remodeling in dogs. The possible mechanisms and optimal time for treatment are discussed.

**Methods:**

We randomly assigned 30 dogs to five groups: RDN 1 week before MI (RDN1w + MI; *n* = 6), RDN 1 week after MI (MI1w + RDN; *n* = 6), RDN 2 weeks after MI (MI2w + RDN; *n* = 6), control (N; *n* = 6), and MI (*n* = 6). A canine model of myocardial infarction was established by interventional occlusion with a gelatin sponge via the femoral artery. Brain natriuretic peptide (BNP) and endothelin-1 (ET-1) levels were measured and echocardiography was performed to assess cardiac function and heart size. All dogs were killed at the end of the experiment and samples of cardiac and renal arteries were obtained. The expression of matrix metalloproteinase (MMP)-2 and MMP-9 in cardiac and of tyrosine hydroxylase (TH) in renal arteries was assessed by immunohistochemistry. Sympathetic innervations in the infarction border zone were investigated via Western blotting and real-time PCR.

**Results:**

Left ventricular function in the MI group decreased significantly, while plasma BNP and ET-1 levels as well as MMP-2 and MMP-9 expression increased. Compared with the MI group, the RD groups showed significantly reduced MMP‑2, MMP‑9, TH, and growth-associated protein (GAP) 43 expression in the RDN1w + MI, MI1w + RDN, and MI2w + RDN groups was significantly improved. Additionally, the expression of TH in renal arteries decreased after RDN.

**Conclusion:**

RDN has preventive and therapeutic effects on post-MI ventricular remodeling and sympathetic neural remodeling. The mechanism of RDN is likely mediated through restraint of renal sympathetic nerve activity.

## Zusammenfassung

**Hintergrund:**

In der vorliegenden Studie wurden die therapeutischen Auswirkungen der renalen sympathischen Denervierung (RDN) auf das ventrikuläre Remodeling nach Myokardinfarkt (MI) und das sympathische neurale Remodeling bei Hunden untersucht. Mögliche Mechanismen und der optimale Zeitpunkt der Behandlung werden erörtert.

**Methoden:**

Randomisiert wurden 30 Hunde auf 5 Gruppen verteilt: RDN 1 Woche vor MI (RDN1w + MI; *n* = 6), RDN 1 Woche nach MI (MI1w + RDN; *n* = 6), RDN 2 Wochen nach MI (MI2w + RDN; *n* = 6), Kontrolle (N, *n* = 6) und MI (*n* = 6). Ein Hundemodell des Herzinfarkts wurde durch interventionelle Okklusion mit einem Gelatineschwamm über die A. femoralis etabliert. Die Werte für BNP („brain natriuretic peptide“) und Endothelin-1 (ET-1) wurden ermittelt und eine Echokardiographie zur Beurteilung der kardialen Funktion und Herzgröße durchgeführt. Alle Hunde wurden am Ende des Experiments getötet und Proben aus den Herz- und Nierenarterien gewonnen. Die Expression von MMP-2 und MMP-9 in Herzarterien und von Tyrosinhydroxylase (TH) in den Nierenarterien wurde immunohistochemisch ermittelt. Sympathische Innervationen in der Infarktgrenzzone wurden per Westernblot und Echtzeitpolymerasekettenreaktion („real-time PCR“) untersucht.

**Ergebnisse:**

Die linksventrikuläre Funktion in der MI-Gruppe nahm signifikant ab, während die Werte für BNP und ET-1 im Plasma sowie für die MMP-2- und die MMP-9-Expression erhöht waren. Im Vergleich zur MI-Gruppe war die linksventrikuläre Ejektionsfraktion, MMP‑2-, MMP‑9-, TH- und „growth-associated protein“(GAP)-43-Expression in den Gruppen RDN1w + MI, MI1w + RDN und MI2w + RDN signifikant verbessert. Außerdem nahm die Expression von TH in den Nierenarterien nach RDN ab.

**Schlussfolgerung:**

Die RDN hat präventive und therapeutische Auswirkungen auf das ventrikuläre Remodeling nach MI und auf das sympathische renale Remodeling. Wahrscheinlich wird der Mechanismus der RDN durch eine Begrenzung der Aktivität des renalen N. sympathicus vermittelt.

Myocardial infarction (MI) is a major cause of death around the world. Although early diagnosis and intervention have improved, approximately one fifth of patients develop ventricular remodeling within 5 years. Ventricular remodeling following MI represents a major cause of morbidity, hospitalization, and premature death. Despite current treatment strategies, MI may result in functional and structural changes in the left ventricle (LV). Ventricular arrhythmia and sudden cardiac death are also major causes of mortality in patients after MI. There is evidence that abnormal sympathetic innervations underlie MI. Sympathoexcitation has long been implicated in the progression of post-MI ventricular remodeling and sympathetic neural remodeling. Excessive sympathoexcitation is associated with lower left ventricular ejection fraction (LVEF) and a higher incidence of ventricular arrhythmia. In the last several years, a novel catheter-based device has become available that specifically interrupts both efferent and afferent renal nerves, termed catheter-based renal sympathetic denervation (RDN). Numerous clinical trials have demonstrated that RDN improves the chronic over-activation of the sympathetic nervous system; furthermore, post-MI remodeling has long been considered a state of generalized sympathetic activation. Thus, we hypothesized that RDN can be used for the treatment of post-MI remodeling and sympathetic neural remodeling.

## Materials and methods

### Animal model

Experiments were carried out using 30 mongrel dogs weighing 15–20 kg, which were purchased through the Experimental Animal Care Center of Tianjin Medical University. All animal experiments followed the Council for International Organization of Medical Sciences (CIOMS) ethical code for animal experimentation and were reviewed and approved by the Animal Use and Management Ethics Committee of Tianjin Medical University.

### Study groups

The dogs were randomly divided into five groups: RDN1w + MI group (RDN 1 week before MI; *n* = 6), MI1w + RDN group (RDN 1 week after MI; *n* = 6), MI2w + RDN group (RDN 2 weeks after MI; *n* = 6), control group (N; *n* = 6), and MI group (*n* = 6).

### Induction of MI

All dogs were anesthetized with pentobarbital (30 mg/kg i.v.), intubated, and ventilated with a respirator with supplemental oxygen. After establishing femoral artery access, a trifle of gelatin sponge was injected distal to the second diagonal branch of the left anterior descending coronary (LAD) as described in a previous study [1], which results in LV mass damage. Lidocaine (1 mg/kg/min i.v. for 70 min, 2 mg/kg i.v. bolus before LAD occlusion) and nitroglycerine (0.5 μg/kg/min i.v. for 70 min starting 10 min before LAD occlusion) were given to decrease the arrhythmia. Animals were observed for 60 min. If ventricular fibrillation occurred, electrical defibrillation was performed immediately.

### Catheter-based renal sympathetic denervation procedures

Dogs were anesthetized with 3% sodium pentobarbital (30 mg/kg) and were placed on the operating table in supine position. The right femoral groin and the back skin area were shaved before connecting the radiofrequency (RF) ablation apparatus (IBI-1500 T, IBI, Abbott, VA, USA). The highest temperature of the RF ablation instrument was 60℃, at a power of 10 W. The area of the operation was disinfected, the right femoral artery was punctured, and a 6-F guiding wire was inserted through the guiding sheath. Renal angiography was performed to determine the location of the renal artery. The ablation electrode (6-F ablation catheter tip, electrode length of 4 mm) was then inserted and RF energy was applied to the endothelial lining. The catheter was subsequently withdrawn by 1–2 cm, circumferentially rotated, and a further dose of RF energy was applied. This procedure was repeated four to six times in the individual renal artery and then the same RF energy was applied to the contralateral renal artery. The target sites were in six different directions, and the ablation procedure lasted for at least 2 min.

### Plasma BNP and ET-1 measurements

Blood samples were collected via jugular venipuncture into chilled glass tubes that contained EDTA and aprotinin for the brain natriuretic peptide (BNP) and endothelin (ET)-1 assays. The samples were centrifuged at 3,000 U/min at 4℃ for 15 min, and the plasma was separated and frozen at −70℃ until analysis. BNP and ET-1 were assayed using enzyme-linked immunosorbent assay (Elisa) techniques that were previously validated for canine plasma samples. All assays were performed in duplicate.

### Evaluation of left atrial structure and function

Echocardiography was performed at baseline and 4 weeks after MI to evaluate cardiac chamber size and LV function (GE VIVID5, Fairfield, CT, USA). Twelve-lead surface electrocardiography (ECG) was also performed at baseline and 4 weeks after MI. LVEF was calculated as (LVVmax − LVVmin)/LVVmax.

### Immunohistochemical studies

Immunohistochemical staining was performed using the Power Vision^TM^ two-step method. Tissues obtained from the LV for immunohistochemical studies were fixed in 4% formalin for 1 h followed by 70% alcohol for more than 48 h. Matrix metalloproteinase-2 (MMP-2), MMP-9, and tyrosine hydroxylase (TH) were stained on 5‑μm sections. The primary antibodies used in this study were polyclonal rabbit anti-MMP-2 antibody (Abcam, MA, USA), polyclonal rabbit anti-MMP-9 antibody (Santa Cruz, CA, USA), and rabbit polyclonal anti-TH antibody (Abcam, MA, USA). The sections were reacted for 20 min with 3% H_2_O_2_ to inactivate endogenous peroxidases; they were then treated with ethylenediaminetetraacetic acid for 10 min at 90℃ in a microwave oven and washed with PBS after being cooled to room temperature. The sections were incubated overnight at 4℃ with primary antibody and with horseradish peroxidase-conjugated second antibody (Santa Cruz, CA, USA). Finally, the sections were thoroughly washed with PBS between each staining. Peroxidase activity was detected using diaminobenzidine. The investigators were blinded to the specimen’s source. We utilized an immunohistochemical score (IHS), which is based on the German ImmunoReactive score; this method has been shown to approximate data generated from image analysis-based scoring systems, as in a previous study [2]. The raw data were converted to the IHS by multiplying the quantity and staining intensity scores.

#### Western blotting

The peri-infarcted zone of the LV and the same zone of the LV in the control group were used for Western blot analysis. First, tissue samples were homogenized on ice in cell lysis buffer; then, they were centrifuged at 10,000 × *g* for 40 min. Equal amounts of proteins were loaded and separated by SDS-PAGE, transferred to a nitrocellulose membrane, and incubated with anti-tyrosine hydroxylase (TH, 1:100, Abcam) and anti-growth-associated protein 43 (GAP43, 1:100, Abcam) overnight at 4℃ followed by incubation with a secondary antibody. The integrated optical densities of these bands were obtained using an imaging system.

#### Real-time PCR

Total RNA was prepared from the peri-infracted border with Trizol reagent (Gibco, USA), and reversely transcribed to cDNA using TaqMan Reverse Transcription Reagents. The expression levels of candidate genes were measured by real-time quantitative RT-PCR using an SYBR Green PCR Master mix. In each assay, both glyceraldehyde-3-phosphate dehydrogenase (GAPDH) and the nerve growth factor (NGF) gene from the same samples were amplified in triplicate in separate tubes. The mRNA levels of NGF were calculated using the relative standard curve method and normalized against the corresponding GAPDH mRNA level, and then expressed as a relative change over the control ± standard deviation (SD). The expected size amplicons were confirmed by gel electrophoreses. The sequences of the genes studied were obtained from GenBank, and the primers were designed using the PRIMER 5.0 software (Applied Biosystems, Foster City, CA, USA). The primer sequence and amplicon size of the genes are shown in Table 1.

**Table 1:** Primer sequence and amplicon size of genes

Gene	Accession no.	Primer and probe sequence	Amplicon size, bp
NGF	NM_001194950.1	F: 5′ AGA CCC GCA ACA TCA CTG TGG 3′	172
		R: 5′ GAA GAC CGC TTG CTC CTG TGA 3′	
β-Actin	NM_001195845.1	F: 5′ ACG GGC AGG TCA TCA CTA TTG 3′	166
		R: 5′ AGC ACT GTG TTG GCA TAG AGG 3′	

### Statistical analysis

Quantitative data are presented as mean ± standard deviation. Group comparisons were made with analysis of variance (ANOVA), followed by the LSD test to identify differences among various groups. SPSS 17.0 was used for the statistical analysis and *p <*0.05 was considered statistically significant.

## Results

In the MI group, two dogs died 1 day after acute MI (AMI) because of arrhythmias (ventricular fibrillation), and one dog died of heart failure 3 weeks after AMI. One dog in the RDN1w + MI group and one in the MI1w + RDN group died 3 days and 1 day after AMI, respectively, because of arrhythmia. In the MI2w + RDN group, one dog died because of arrhythmia and one died of heart failure. In the control group, no death occurred.

### Effect of RDN on changes in plasma BNP and ET-1

**Fig. 1:**
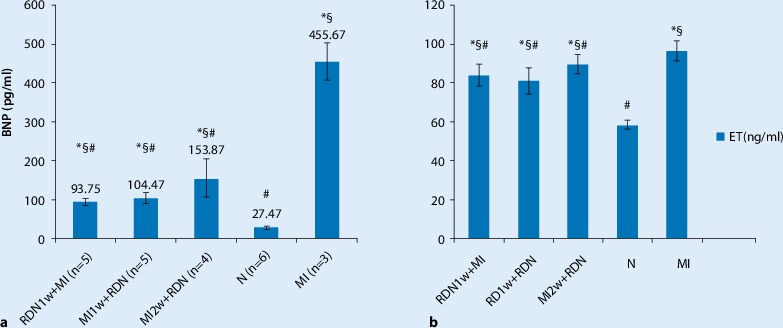
Level of (**a**) brain natriuretic peptide (*BNP*) and of (**b**) endothelin-1 (*ET-1*) 4 weeks after myocardial infarction (*MI*).Data are mean ± SD obtained from baseline and 4 weeks after MI. ^*§*^*p* < 0.05: group comparison; ^*^p *< *0.05 vs. N group; ^#^*p* *< *0.05 vs. MI group

The level of plasma BNP and ET-1 in the RDN groups (including the RDN1w + MI group, the MI1w + RDN group, and the MI2w + MI group) decreased significantly (*p* <0.05) compared with the MI group, and it was significantly increased compared with the N group (*p* <0.05). There was no significant difference between the RDN1w + MI group and the MI1w + RDN group, but the level of BNP in both groups decreased compared with that in the MI2w + MI group; the level of BNP was higher in RDN and MI groups compared with that in the N group (*p* <0.05; Fig. 1).

### Changes in atrial structure and function

**Fig. 2:**
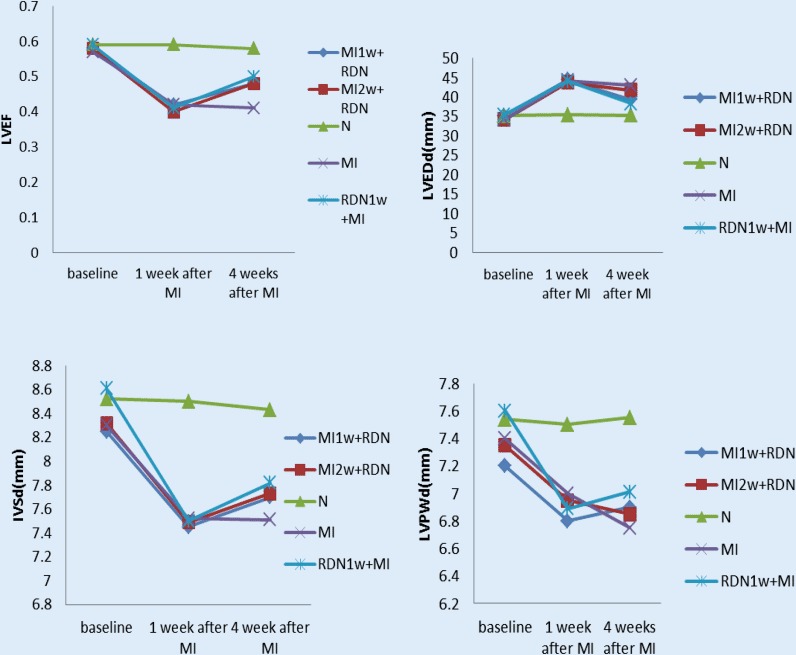
Left ventricular ejection fraction (*LVEF*), interventricular septal thickness (*IVSd*), left ventricular posterior wall thickness (*LVPWd*), and left ventricular end-diastolic diameter (*LVEDd*) measured at baseline, 1 week after MI, and 4 weeks after MI in the five study groups: *RDN1w* *+MI* RDN 1 week before MI, *MI1w* *+RDN*, RDN 1 week after MI, *MI2w* *+RDN *RDN 2 weeks after MI, *N* control group, *MI* myocardial infarction group (see text for details)

There was no significant difference among all groups at baseline (all *p* > 0.05). Echocardiography was performed for the second time 1 week after MI, and showed no difference among the RDN1w + MI, MI1w + RDN, MI2w + RDN, and MI groups (all *p* > 0.05). Moreover, 1 week after MI, LVEF, interventricular septal thickness (IVSd), and left ventricular posterior wall thickness (LVPWd) in the MI group were significantly reduced (all *p < *0.05), while left ventricular end-diastolic diameter (LVEDd) was significantly increased. After RDN (4 weeks after MI, echocardiography was performed for the third time), the LVEF, IVSd, and LVPWd in the RDN groups (RDN1w + MI, MI1w + RDN, MI2w + RDN) had significantly increased compared with the MI group, and had decreased compared with the N group; however, there was no difference between the MI2w + RDN group and the MI group in terms of IVSd. The LVEDd in the RDN groups had significantly decreased compared with the MI group and had increased compared with the N group (Table 2 and Fig. 2).

**Table 2:** Changes in atrial structure and function

Groups	LVEF	LVEDd(mm)	IVSd(mm)	LVPWd(mm)
RDN1W + MI group	0.5 ± 0.05^*§#^	38.4 ± 3.79^*^^§^^#^	7.82 ± 1.63^*^^§^^#^	7.01 ± 1.15^*^^§^^#^
MI1w + RDN group	0.48 ± 0.05^*^^§^^#^	39.4 ± 3.02^*§#^	7.70 ± 1.41^*§#^	6.60 ± 0.82^*§#^
MI2w + RDN group	0.48 ± 0.06^*§#^	41.8 ± 2.51^*§#^	7.73 ± 1.43^*§^	6.85 ± 0.95^*§#^
N group	0.58 ± 0.03^#^	35.3 ± 2.49^#^	8.43 ± 1.59^#^	7.55 ± 1.10^#^
MI group	0.41 ± 0.05^*§^	43.0 ± 2.61^*§^	7.51 ± 1.63^*§^	6.75 ± 1.05^*§^

Data are mean ± SD obtained from baseline and 4 weeks after MI

*LVEF* left ventricular ejection fraction, *LVEDd* left ventricular end-diastolic diameter, *IVSd* interventricular septal thickness, *LVPWd* left ventricular posterior wall thickness, *MI* myocardial infarction, *RDN* renal sympathetic denervation, *RDN1w* *+MI* RDN 1 week before MI, *MI1w* *+RDN*, RDN 1 week after MI, *MI2w* *+RDN *RDN 2 weeks after MI, *N* control group

^§^*p* *<* 0.05: group comparison, ^*^p *<* 0.05 vs. N group, ^#^*p* *<* 0.05 vs. MI group

### Changes in TH-positive nerve fibers after RDN

**Fig. 3:**
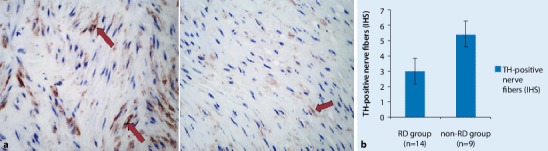
**a** Tyrosine hydroxylase (*TH*) nerve fiber staining. **a** non-RDN groups (*left*): the distribution of TH-positive nerve fibers is thickened and disorderly (*arrows*); RDN groups (*right*): TH-positive nerve fibers in renal artery are significantly decreased with orderly distribution (*arrow*). **b** Expression of TH-positive nerve fibers in the renal artery. *IHS* immunohistochemical score. ×400

After RDN, we found that the expression of TH-positive nerve fibers in the renal artery of the RDN group (RDN1w + MI, MI1w + RDN, MI2w + RDN) was significantly reduced compared with the non-RDN group (N group and MI group; *p* <0.05; Fig. 3).

### Effect of RDN on MMP-2 and MMP‑9 expression

**Fig. 4:**
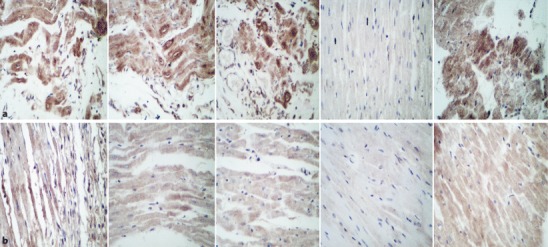
**a** Matrix metalloproteinase (MMP)-2 and **b** MMP-9 immunohistochemical staining of the infarct border zone. ×400. *From left to right*: RDN1w + MI group, MI1w + RDN group, MI2w + RDN group, N group, and MI group (see text for details)

**Fig. 5:**
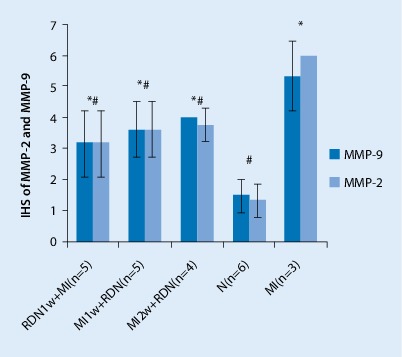
Immunohistochemical score (*IHS*) for matrix metalloproteinase-2 (*MMP-2*) and matrix metalloproteinase-9 (*MMP-9*) expression. Data are mean ± SD obtained 4 weeks after MI. *p < 0.05 vs. N group, #*p* < 0.05 vs. MI group (see text for details)

Through immunohistochemical staining and by calculating the immunohistochemical score (IHS), we found the expression of MMP-2 and MMP-9 in the LV to be significantly reduced in the RDN1w + MI, MI1w + RDN, and MI2w + RDN groups compared with the MI group, and to be significantly increased compared with the N group. However, there was no difference among the RDN1w + MI, MI1w + RDN, and MI2w + RDN groups (Figs. 4 and 5).

### TH and GAP43 protein expression

**Fig. 6:**
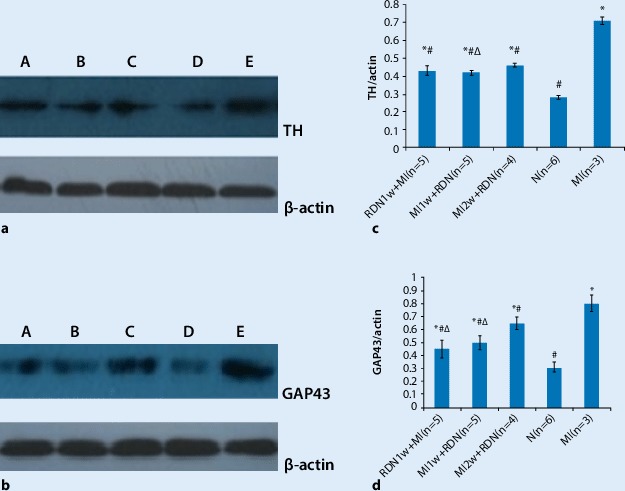
**a** Western blots and graphs showing protein expression of (**a, c**) tyrosine hydroxylase (*TH*) and β-actin as well as of (**b, d**) GAP43 and β-actin in the peri-infarct zone (**c,** **d** mean ± SD). *A* RDN1w + MI group, *B* MI1w + RDN group, *C* MI2w + RDN group, *D* N group, *E* MI group

In our study, the protein expression levels of TH and GAP43 were significantly lower in the RDN groups (RDN1w + MI, MI1w + RDN, MI2w + RDN) than in the MI group, but higher than in the control group. RDN treatment significantly reduced the protein expression of TH and GAP43. Moreover, the protein expression of TH and GAP43 in the RDN1w + MI and MI1w + RDN groups was lower than in the MI2w + RDN group, but there was no difference between the RDN1w + MI and MI1w + RDN groups (Fig. 6).

### Effect of RDN on NGF expression

**Fig. 7:**
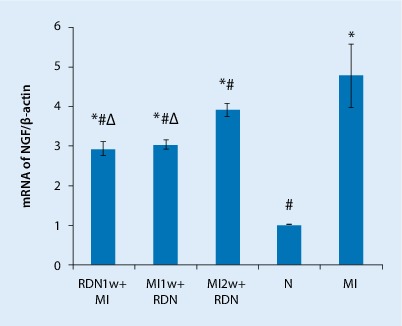
mRNA levels of nerve growth factor (*NGF*) quantified in the peri-infarct zone. Data are mean ± SD obtained from baseline and 4 weeks after MI. ^*△*^*p* *< *0.05 vs. MI2w + RDN group, ^*^p *< *0.05 vs. N group, ^#^*p* *< *0.05 vs. MI group (see text for details)

In line with the protein expression, RDN therapy resulted in significant decreases in mRNA expression. The relative NGF mRNA levels in the peri-infarct area are shown in Fig. 7. The mRNA expression levels of NGF were significantly lower in the RDN group (RDN1w + MI, MI1w + RDN, MI2w + RDN) than in the MI group, but higher than in the control group. RDN treatment significantly reduced the mRNA expression of NGF. Furthermore, the mRNA expression of NGF in the RDN1w + MI and MI1w + RDN groups was lower than in the MI2w + RDN group; however, there was no difference between the RDN1w + MI and MI1w + RDN groups (Fig. 7).

## Discussion

MI is commonly complicated by maladaptive LV remodeling, which refers to alterations in LV chamber mass, geometry, and function. Remodeling is a chronic process, mediated by progressive structural changes in cardiomyocytes and the extracellular matrix (ECM) and by interstitial fibrosis, leading to LV dilation. ECM turnover is involved in LV remodeling. Various members of the MMP family have been described to mediate post-MI LV remodeling [3, 4]. Specifically, MMP-9 and MMP-2 are recognized as major contributors. The increased cardiac activity of MMP-2 and MMP-9 was associated with ventricular remodeling in several experimental studies. Targeted deletion of MMP-9 and MMP-2 results in significantly reduced LV enlargement following MI [5].

LV dysfunction triggers alterations of many mediators such as neurohumoral factors, cytokines, enzymes, ion channels, oxidative stress, and mechanical stress [6], thereby promoting cardiac remodeling. Tsuruda et al. reported that BNP induces protein expression of MMPs, suggesting that stimulation of MMPs by BNP may be a compensatory response to prevent excessive collagen deposition induced by profibrotic factors [7]. BNP plays important roles in maintaining cardiorenal homeostasis under physiological and pathological conditions. BNP is a marker of congestive heart failure, its levels paralleling the degree of LV remodeling [8, 9]. BNP is synthesized by cardiomyocytes, and their production is stimulated in pathologic conditions such as MI. BNP and its signaling system contribute to the regulation of collagen synthesis and to the activation of MMPs. In addition, ET-1 also activates the generation of MMPs.

Several studies support the concept that direct recordings of sympathetic nerve activity in animal models are likely to reveal mechanisms producing sympathoexcitation in post-MI ventricular remodeling. It has been postulated that the increase in cardiac sympathetic nerve activity is the most damaging aspect of the sympathoactivation in heart failure [10]. The increase in sympathetic nerve activity causes the expression of MMPs to increase.

Animal studies showed that inhibition of the sympathetic nervous system could confer protection against the damage to organs due to chronic excessive activation of sympathetic nerves [11, 12]. In recent years, RDN treatment of hypertension and diseases caused by chronic hyperactivity of the sympathetic nervous system (such as heart failure, hypertension accompanied by impaired glucose tolerance, left ventricular hypertrophy, and chronic renal dysfunction) has been a hot research topic in the field [13–16]. Whole-body norepinephrine spillover of 42% and efferent muscle sympathetic nerve activity of 66% were reported after RDN. Thus, blocking of the sympathetic nervous system by RDN may be an important component in the prevention of ventricular remodeling and improvement of LV function after MI.

However, in our study we found that RDN improved not only ventricular remodeling, but also sympathetic neural remodeling. Increased sympathetic activity constitutes an important factor in the genesis of post-MI ventricular arrhythmias. β‑Adrenergic blocking agents are the mainstay in the prophylaxis of ventricular arrhythmias and sudden cardiac death. Ventricular tachycardia and fibrillation are major causes of morbidity and mortality in patients with MI. Previous studies have shown that heterogeneous cardiac nerve sprouting and sympathetic hyperinnervation, which is called sympathetic neural remodeling, contribute to ventricular arrhythmogenesis and sudden cardiac death in patients and animal models of MI alike. Strategies aiming at reducing sympathetic activity potentially protect against ventricular arrhythmias.

MI is associated with elevated NGF levels, and NGF protein and mRNA are localized primarily within macrophages and myofibroblasts. TH is the rate-limiting enzyme of NE synthesis, which serves not only as a marker of sympathetic nerve terminals but also as an indirect indicator of sympathetic activity. The combination of GAP43 and TH can precisely reflect the sprouting of sympathetic nerves. In addition, sympathetic sprouting and sympathetic remodeling reach a peak at 1 week after MI. MI-induced inflammation upregulates NGF, which is critical for sympathetic sprouting and is spatiotemporally consistent with sympathetic hyperinnervation. In our study, we found that RDN modulates the expression of TH, GAP43, and NGF after MI. This means that RDN inhibited sympathetic neural remodeling by decreasing TH, GAP43, and NGF expression in peri-infarcted hearts.

The present study demonstrated that RDN, whether performed before or after MI, prevented and improved the post-MI deterioration of LV function and LV dilatation; furthermore, LVEF increased significantly, while LVEDd decreased significantly compared with the MI group, as found in the study of Hu et al. [17]. In our study, we also demonstrated that RDN reduced the level of BNP and ET-1 as well as the expression of MMP-2, MMP-9, TH, GAP43, and NGF. We found a decrease in the amount of sympathetic nerve activity around the renal artery after RDN.

Zozawa et al. observed that RDN prior to MI in rats improved cardiac performance [18]. In particular, the RDN group had lower end-diastolic pressures, greater fractional shortening, and improved sodium excretion compared with the intact group. The authors speculated that the positive actions of RDN were through improved renal function and reduced angiotensin II. Sato et al. [19] reported that in tachycardia-induced heart failure, dogs with cardiac sympathetic denervation tolerated the development of heart failure better than intact dogs without denervation and had significantly less catecholamine desensitization.

Previous results suggest that long-term RDN inhibits ventricular dilatation after MI, probably due to the improvement in natriuresis. In our study, we found similar results—the level of plasma BNP decreased after RDN. Based on our previous study, we found that RDN also blocked the renin-angiotensin-aldosterone system (RAAS; [20–22]) and improved the vascular endothelin system, e.g., reduced ET-1. Therefore, RDN may lessen the effects of angiotensin II and aldosterone on ventricular structure and function.

Moreover, RDN reduced the expression of MMP-2, MMP-9, TH, GAP43, and NGF, it lessened the ability of cardiomyocytes to degrade ECM proteins, and attenuated early rupture and improved cardiac function after MI; furthermore, RDN decreased the distribution of sympathetic neural in the peri-infarct zone and it improved sympathetic neural remodeling.

MI leads to progressive LV dilatation and ventricular arrhythmia. The NF-κB signaling pathway plays an important role in ventricular remodeling and sympathetic neural remodeling after MI. Activation of NF-κB induces genetic programs that lead to the transcription of cytokines, chemokines, MMPs, and NGF, promoting inflammatory and fibrotic responses that participate in the progression of ventricular remodeling [23, 24] and sympathetic neural remodeling. Jiang et al. found that RDN compromises the immune/inflammatory response, reducing the protein expression of NF-κB, which may further improve cardiac function and decrease the incidence of ventricular arrhythmia; this also suggests that inhibition of the expression of local NF-κB may be the mechanism underlying the RDN-reduced expression of MMPs and NGF.

RDN 1 week before MI may decrease the excessive sympathetic response in the acute phase of MI. However, in our study, we did not find any differences between the RDN1w + MI group, the MI1w + RDN group, and the MI2w + RDN group. The mechanism is not very clear. We infer that post-MI remodeling is a long-term process, including early ventricular remodeling and late ventricular remodeling. Early ventricular remodeling mainly occurs 6 weeks after MI. In our experiments, the timing of RDN was too close to find any difference. However, there were some differences in post-infarct sympathetic neural remodeling; the expression of TH, GAP43, and NGF in the MI2w + RDN group was higher than that in the RDN1w + MI group and in the MI1w + RDN group. The reason for this is that sympathetic sprouting and sympathetic remodeling reach a peak at 1 week after MI; 2 weeks after MI, the sympathetic neural remodeling in the peri-infarct zone was higher than that in the RDN1w + MI group and in the MI1w + RDN group.

### Limitations

Some limitations should be considered in the present study. First, the sample size was not large enough, which may have affected statistical analysis. Second, the lack of an N + RDN group (RDN in normal dogs) cannot rule out the effects of intervention completely. Third, the observation time was short.

## Conclusion

RDN can protect and improve post-MI ventricular remodeling by reducing the level of plasma BNP and ET-1 and by decreasing the expression of MMP-2 and MMP-9. Most importantly, RDN can improve post-MI sympathetic neural remodeling by reducing the expression of TH, GAP43, and NGF.
